# Prostate MRI cancer detection rate by deep learning-assisted image quality categorization: gas-induced susceptibility artifacts in diffusion-weighted imaging

**DOI:** 10.1186/s13244-025-02110-6

**Published:** 2025-10-15

**Authors:** Hirotsugu Nakai, Adam T. Froemming, Hiroaki Takahashi, Daniel A. Adamo, Akira Kawashima, Jordan D. LeGout, Yasuhisa Kurata, Jacob N. Gloe, Eric A. Borisch, Stephen J. Riederer, Naoki Takahashi

**Affiliations:** 1 Department of Radiology, Mayo Clinic, Rochester, MN USA; 2 Department of Radiology, Mayo Clinic, Scottsdale, AZ USA; 3 Department of Radiology, Mayo Clinic, Jacksonville, FL USA

**Keywords:** Magnetic resonance imaging, Prostate cancer, Deep learning, Artifacts, Image quality enhancement

## Abstract

**Objectives:**

To evaluate the impact of gas-induced artifacts in diffusion-weighted imaging (DWI) on prostate MRI cancer detection rate (CDR).

**Methods:**

This three-center retrospective study included 34,697 MRI examinations between 2017 and 2022. Seven radiologists categorized the degree of gas-induced artifacts of 1595 DWI series into optimal, mild, moderate, and severe. Then, a deep learning model categorizing artifact severity was developed to help identify series with gas-induced artifacts. After excluding series used for training the model, the model was applied to 12,594 DWI series, which were performed for patients without documented prostate cancer. Of these, radiologists reviewed the bottom 300 series predicted as poor image quality and recategorized them if necessary. Case-control matching was performed to compare CDR. Examinations categorized by radiologists as mild-severe were used as target groups, while those categorized as optimal by either radiologists or the model were used to construct matched control groups. CDR was defined as the number of examinations assigned PI-RADS ≥ 3 with pathologically proven clinically significant cancer divided by the total number of examinations. The degree of CDR reduction was evaluated using the chi-squared test.

**Results:**

The target groups included 632 examinations (66.0 ± 9.5 years). The CDR in the target and matched control groups, respectively, for each artifact grade were as follows: severe (*n* = 141) vs optimal (*n* = 705), 0.24 vs 0.26, *p* = 0.58; moderate (*n* = 161) vs optimal (*n* = 966), 0.25 vs 0.24, *p* = 0.84; mild (*n* = 330) vs optimal (*n* = 1320), 0.25 vs 0.22, *p* = 0.17.

**Conclusion:**

No evidence was found that gas-induced DWI artifacts reduce the CDR of prostate MRI.

**Critical relevance statement:**

The CDR of prostate MRI was not significantly reduced by susceptibility artifacts from rectal gas, which will be one consideration in rectal preparation protocols.

**Key Points:**

- Gas-induced susceptibility artifact is a common issue in prostate MRI.
- The CDR decreased as the degree of artifacts increased.
- But there was no significant reduction even in severe artifact cases.

**Graphical Abstract:**

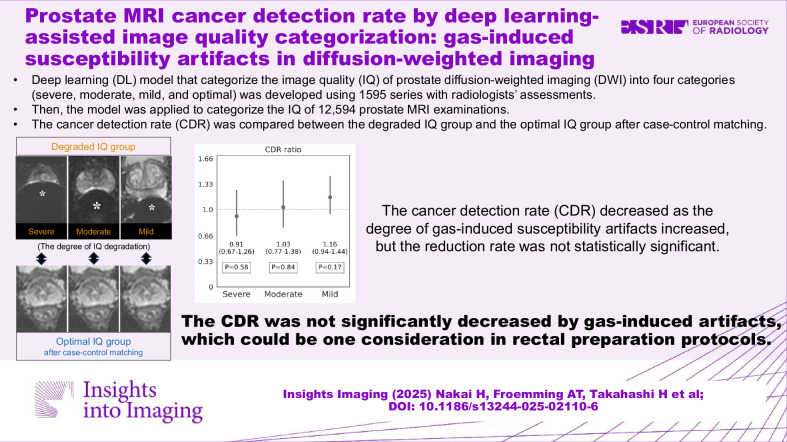

## Introduction

MRI is the standard imaging technique for the prostate and plays an important role in detecting and avoiding overdiagnosis of clinically significant prostate cancer (csPCa) [1–3]. High-quality images are prerequisites for appropriate interpretation and subsequent management decisions [4, 5]. To standardize image quality (IQ) assessment, the prostate image quality (PI-QUAL) scoring system has been proposed, which summarizes the overall IQ across three sequences [6–8]. Though a limited number of studies have evaluated the impact of poor-quality images on csPCa diagnosis, some have reported that examinations with low PI-QUAL scores may result in more false-positive calls and unnecessary biopsies due to increased diagnostic uncertainty [9, 10]. Given the differences in possible countermeasures, it is desirable to clarify the impact of each type of IQ degradation. One of the key factors defining IQ is the presence of significant artifacts.

Diffusion-weighted imaging (DWI) is one of the most important sequences in prostate MRI [11] and the most vulnerable to susceptibility artifacts [12]. Susceptibility artifacts are common challenges in interpretation, and their two main sources are hip prostheses and rectal gas. One study showed that DWI with moderate-severe susceptibility artifacts from hip prostheses reduced cancer detection rate (CDR) by 74% compared to those without hip prostheses [13]. Susceptibility artifacts from rectal gas are even more common than hip prostheses and affect mostly the peripheral zone, where csPCa is more prevalent [14, 15]. However, the amount of artifacts from gas is usually less than that from hip prostheses. The clinical impact of gas-induced artifacts on diagnosing csPCa remains unclear. We sought to clarify this impact to inform the need for countermeasures, such as a pre-scan enema or rescan with an alternative diffusion-encoded sequence. For analyzing the impact of gas-induced artifacts, we need another IQ evaluation metric different from PI-QUAL, which roughly evaluates the presence of all types of artifacts.

Qualitative IQ assessment is highly subjective and can be influenced by experience [16]. To make the criteria more objective, incorporating numerical values may be considered. Additionally, deep learning (DL) potentially provides consistent and more objective IQ assessments that can mimic expert radiologists [17–19]. DL-based assessments may be integrated into clinical workflow to provide a swift and impartial assessment.

This study aimed to compare the CDR of prostate MRI between groups with different degrees of gas-induced DWI susceptibility artifacts and those without.

## Materials and methods

This three-center retrospective study was approved by our institutional review board with an informed consent waiver (#23-008038). Thousands of patients overlapped with prior publications on prostate MRI [13, 20–26].

### Prostate MRI

There were 34,697 bi- or multi-parametric prostate MRI examinations from 2017 to 2022 (Population-A). Most were performed without endorectal coils using 3-T scanners (GE HealthCare or Siemens). Contrast material and glucagon were used unless contraindicated. Other rectal preparation protocols varied across facilities (I, none; II, enema instruction at home; III, defecation instruction before scanning). In most DWI sequences, reduced volume excitation techniques (FOCUS or Zoomit) were used, and computed DWI with *b*-values of 1400 s/mm^2^ was created. Board-certified, fellowship-trained abdominal radiologists reported all examinations using the same structured report template based on PI-RADS v2.0 or v2.1 criteria. 99% of the reports were read by radiologists with over 100 prostate MRI interpretations per year.

### Overview

This study largely consists of two sections: (A) development and evaluation of an IQ classification model using a subset of the data, and (B) comparing CDR per model-assisted IQ categorization for the entire prostate MRI population performed with clinical suspicion of csPCa. For each section, the data and analysis were described separately.

### (A) IQ classification model

#### DL dataset

**Fig. 1:**
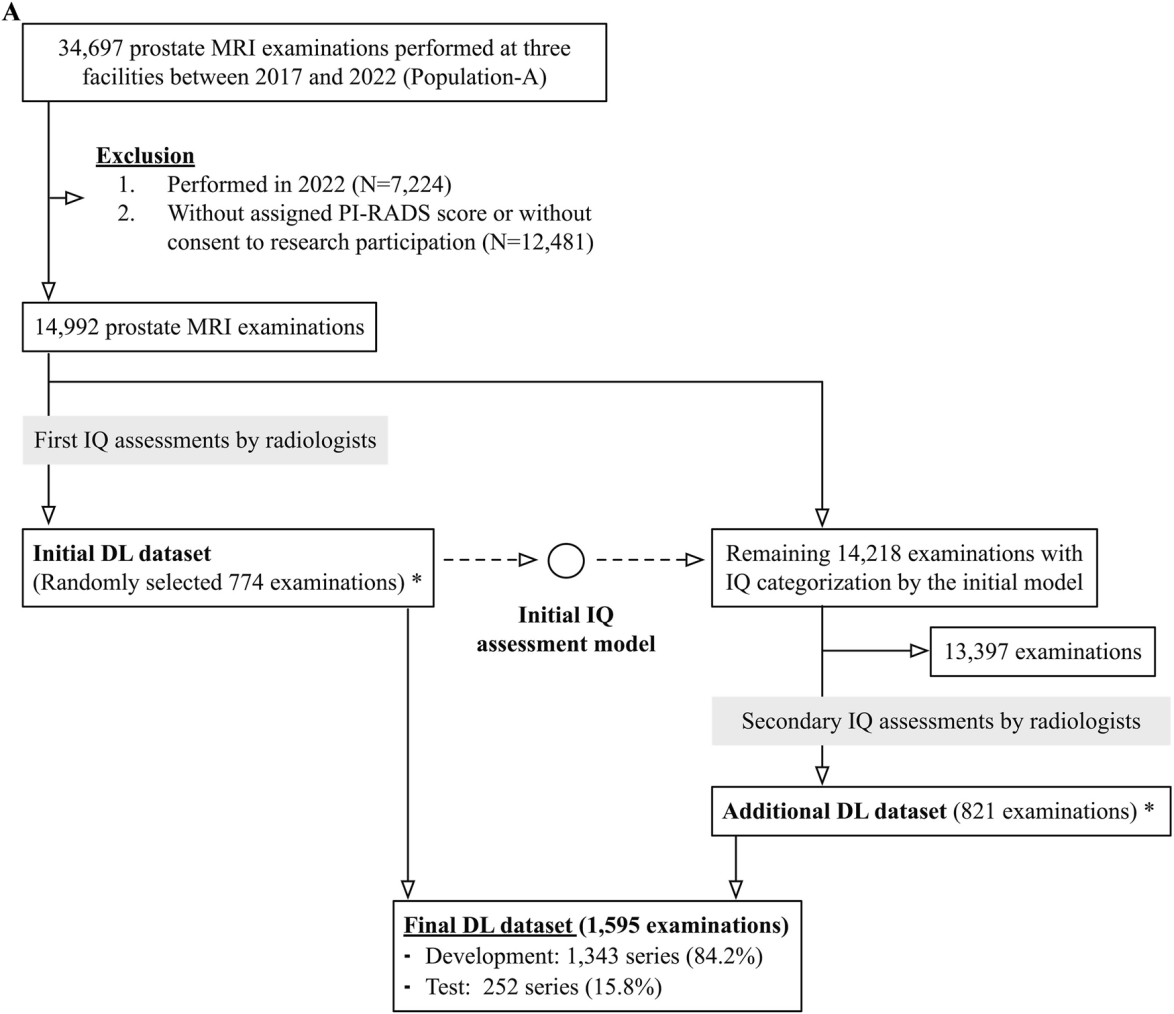

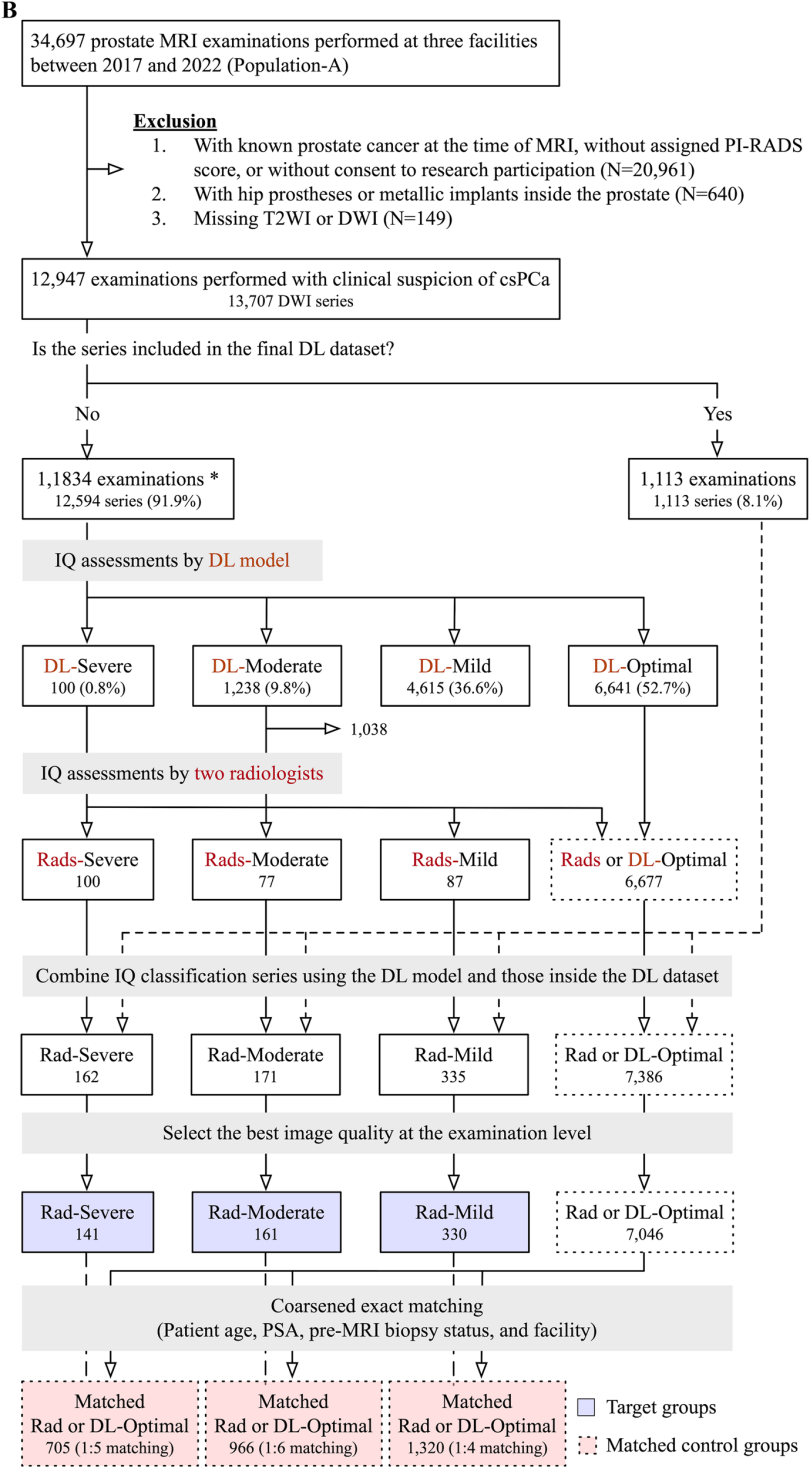
Flowcharts: population A in **A** and **B** refers to the same cohort. **A** DL dataset. The initial and final DL datasets contained a single series per examination. At the time of dataset creation, examinations conducted in 2022 were unavailable. * The initial DL dataset was also used to assess patient-related factors associated with IQ categorized by radiologists (Table 3). **B** Examinations to evaluate the diagnostic performance by the degree of susceptibility artifact; severity categorization, image selection, and matching. The numbers inside the boxes represent the number of series of DWI. Blue- and red-highlighted boxes represent the target groups and matched control groups, respectively. * Examinations not used in the DL dataset were also used to assess patient-related factors associated with the degree of susceptibility artifacts categorized by models (Table 3). csPCa, clinically significant prostate cancer; DL, deep learning; DWI, diffusion-weighted imaging; IQ, image quality; PI-RADS, prostate imaging-reporting and data system; PSA, prostate specific antigen; Rads, radiologists; T2WI, T2-weighted imaging

**Fig. 2:**
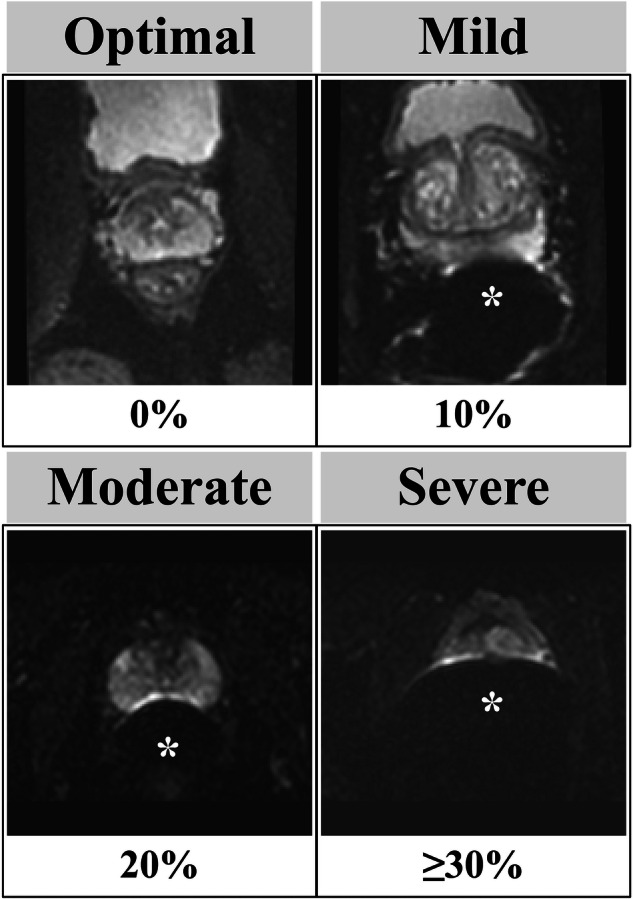
Degree of susceptibility artifact examples. The degree of gas-induced susceptibility artifacts in low b-value axial diffusion-weighted images (≤ 100 s/mm^2^) was categorized by the obscured prostate proportion, given as a percentage. The * indicates the susceptibility artifact from rectal gas

First, seven abdominal radiologists, each with ≥ 5 years of post-training experience and over 100 prostate MRI interpretations per year, reviewed 774 randomly selected series (the initial dataset, 5.2%) from 14,992 examinations with PI-RADS scores performed before 2021 (Fig. 1). Low *b*-value axial DWI (≤ 100 s/mm^2^) was used to see the prostate boundary easily. The prostate proportion obscured by gas was scored in 10% increments and was converted into severe (≥ 30%), moderate (20%), mild (10%), and optimal (0%) (Fig. 2). Next, a preliminary DL model was developed and applied to the remaining 14,218 (94.8%) examinations. Using their predictions, the radiologists similarly reviewed an additional 821 series focusing on relatively uncommon poor-quality categories (i.e., moderate-severe). To minimize potential prediction bias, the breakdown of the PI-RADS score, facility, and examination year per each IQ category was adjusted as closely as possible. The distribution of the IQ in the additional 821 series is as follows: severe, 58 (7.1%); moderate, 70 (8.5%); mild, 208 (25.4%); optimal, 485 (59.1%).

The final dataset (*N* = 1595) was divided into a development set (*N* = 1343, 84.2%) and a test set (*N* = 252, 15.8%). Multiple radiologists reviewed each examination in the test set, and their median category was used to compare with the model. In 24 out of the 252 cases (9.5%) where the median was not on the four-point scale, one radiologist (N.T.) made the final categorization.

#### Development

**Fig. 3:**
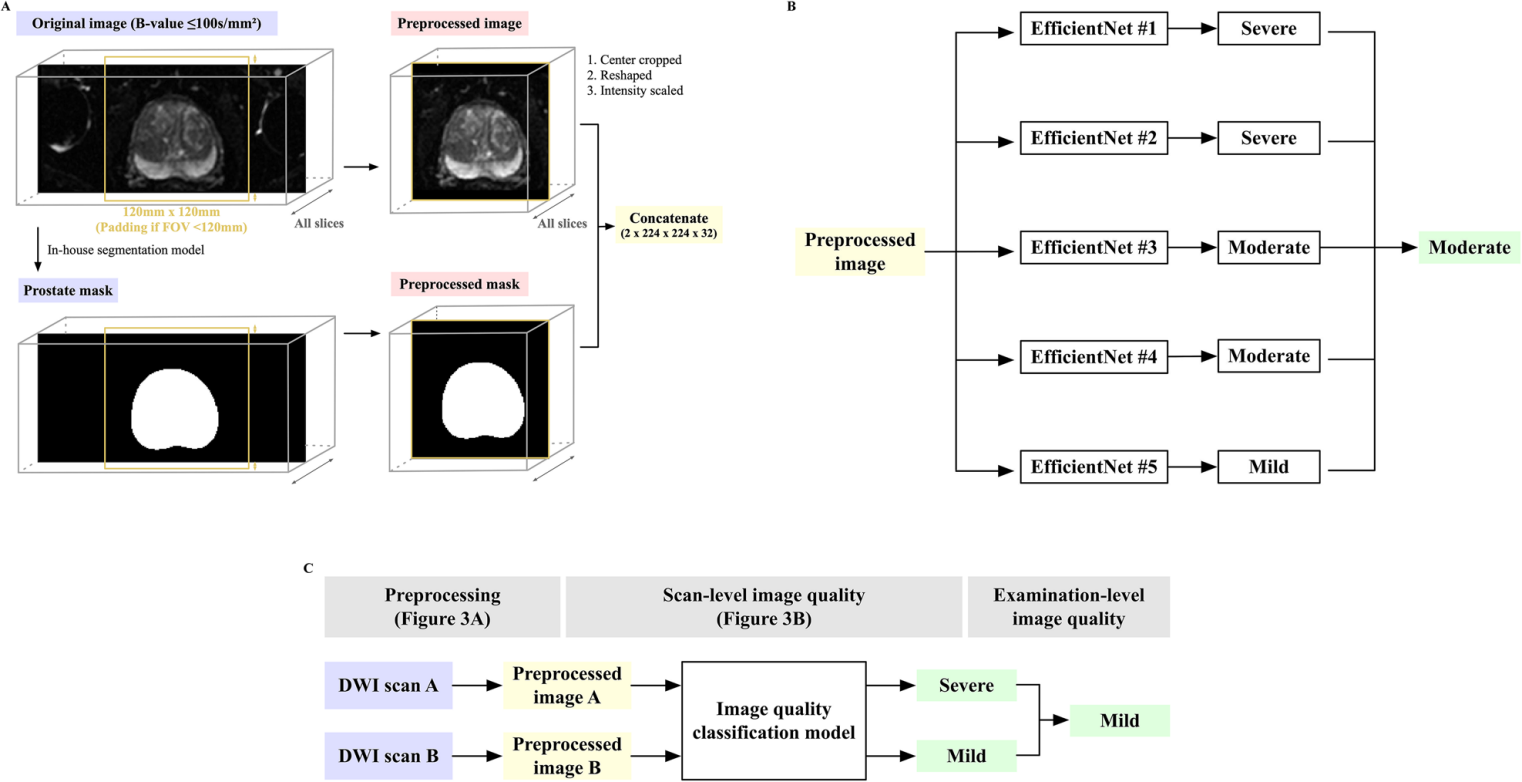
Image quality (IQ) assessment model. **A** Preprocessing: this figure illustrates the preprocessing in the IQ assessment model. First, a prostate mask was created from the original DWI using an in-house segmentation model. Next, the original image and prostate mask were center-cropped (120 mm × 120 mm) and reshaped (1 × 224 × 224 × 32). Then, their voxel intensity was scaled to a minimum of 0 and a maximum of 1. Finally, the preprocessed images were concatenated. **B** Example of the model’s output. **C** Example of selecting the best IQ at the examination level. DWI, diffusion-weighted imaging; FOV, field of view

The classification model was an ensemble of five sub-models; each utilized 3D EfficientNet-B0 [27] and underwent training using five-fold cross-validation within the development set (see Appendix S1). Based on hyperparameter tuning, an in-house segmentation model (Dice coefficient: 0.96 ± 0.03, *N* = 20, unpublished data) was utilized. Each sub-model used a center-cropped image and a corresponding prostate mask as inputs; radiologists’ quality categorization served as output. The model returned the median of the five outputs (Fig. 3A, B).

#### Evaluation on the test set

Agreement among seven radiologists was assessed with multi-rater generalized kappa [28] using 37 randomly selected examinations reviewed by all. Models’ predictions against the radiologists were shown using a confusion matrix with quadratic weighted kappa [28]. The agreements between the model and radiologists were categorized using kappa statistics as follows: 0.81–1.00, almost perfect; 0.61–0.80, substantial; 0.41–0.60, moderate; 0.21–0.40, fair; 0.00–0.20, slight; and below 0.00, poor [28].

### (B) CDR comparison

#### Population

The following examinations were excluded from Population A: known prostate cancer (Grade group ≥ 1) at the time of MRI, presence of hip prostheses or metallic prostate implants, or missing T2-weighted imaging (T2WI) or DWI. The remaining 12,947 examinations (13,707 DWI series) performed with clinical suspicion of csPCa were included. The model was applied to all 12,594 (91.9%) DWI series not used in the DL dataset.

The results of the previous section (A), which will be described later, found that the model’s positive predictive value (PPV) in poor-quality categories was not high enough to assess the impact of degraded IQ. Therefore, to identify more poor-quality series with appropriate categories, two radiologists (H.N. and N.T.), who also reviewed the DL dataset, evaluated all series predicted as severe and 200 series as moderate but closest to severe. Then, these radiologists’ assessments were combined with the DL dataset. For examinations with multiple DWI series, the best series-level IQ was used as the examination-level IQ (Fig. 3C).

#### Matching

Examinations categorized by radiologists as mild, moderate, or severe artifacts were defined as the target groups. In contrast, those classified as optimal by either radiologists or the model served as the control pool. Matched control groups were constructed using a repeated coarsened exact matching procedure [29]. Potential confounders adjusted for included patient age, prostate-specific antigen (PSA) value, pre-MRI biopsy status, and facility. Age and PSA were categorized into 3 and 6 equal-sized bins, respectively (wider and narrower bins), with an additional bin assigned to missing PSA values.

Matching was performed iteratively in multiple rounds. In each round, one candidate control examination was searched for each target examination, prioritizing exact matches within the narrower bins; if no match was available, wider bins were used. When multiple candidate matches were found, a single control examination was selected randomly. A round was considered valid only if every target examination was successfully matched; otherwise, the round was discarded and did not contribute to the matched set. Once matched, the control examination was removed from the pool to prevent reuse. The matching was performed independently for each artifact category. Thus, the same control patient could be included in more than one artifact category.

#### Clinical data

Pathological diagnosis within one year after the MRI was used to diagnose csPCa (Grade group ≥ 2). Pre-MRI biopsy status was extracted and categorized into biopsy naive, previous benign biopsy, and unknown using an in-house natural language processing pipeline [22]. A trans-rectal or trans-perineal ultrasound-guided targeted biopsy was performed by urologists using fusion software (UroNav, Philips Healthcare). Systematic biopsies (10–12 cores) were performed simultaneously. If clinically indicated, systematic biopsies were performed for PI-RADS 1–2 cases. The PSA value was extracted from MRI reports.

#### Primary analysis

Primary analysis compared CDR, abnormal interpretation rate (AIR), PPV, and pathological confirmation rates between the target groups and the matched control groups by the degree of susceptibility artifacts. The CDR and AIR are recently proposed metrics for prostate MRI [20, 21] and can be applied to the entire prostate MRI population regardless of the presence of pathological confirmation of the prostate. Their definitions were as follows:

$${{CDR}}= 	 \frac{{{number}}\,{{of}}\,{{exams}}\,{{with}}\,({{csPCa}}\,{{\cap} }{{PI}}\mbox{-}{{RADS}}\ge 3)}{{{total}}\,{{number}}\,{{of}}\,{{exams}}} \\ \\ {{AIR}}= 	 \frac{{{number}}\,{{of}}\,{{exams}}\,{{with}}\,({{PI}}\mbox{-}{{RADS}}\ge 3)}{{{total}}\,{{number}}\,{{of}}\,{{exams}}}\\ \\ {{PPV}}= 	 \frac{{{number}}\,{{of}}\,{{exams}}\,{{with}}\,({{csPCa}}\cap {{PI}}\mbox{-}{{RADS}}\ge 3)}{{{number}}\,{{of}}\,{{exams}}\,{{with}}\,({{any}}\,{{pathological}}\,{{diagnosis}}\cap {{PI}}\mbox{-}{{RADS}}\ge 3)} \\ \\ {{Pathological}}\,{{confirmation}}\,{{rate}} = 	 \frac{{{number}}\,{{of}}\,{{exams}}\,{{with}}\,({{any}}\,{{pathological}}\,{{diagnosis}}\cap {{PI}}\mbox{-}{{RADS}}\ge 3)}{{{number}}\,{{of}}\,{{exams}}\,{{with}}{{PI}}\mbox{-}{{RADS}}\ge 3}$$

Ratios of these metrics were calculated by dividing the value in the target group by that in the matched control group. The CDR (ratio) is a product of AIR, PPV, and pathological confirmation rate (ratios), which were used to assess the contribution to change in the CDR (ratio).

$${{CDR}}= 	 \,\frac{{{number}}\,{{of}}\,{exams}\,{{with}}\,({{csPCa}}\cap {{PI}}\mbox{-}{RADS}\ge 3)}{{total}\,{{number}}\,{{of}}\,{exams}}\\ = 	 \frac{{{number}}\,{{of}}\,{{exams}}\,{{with}}\,{{PI}}\mbox{-}{{RADS}}\ge 3}{{{total}}\,{{number}}\,{{of}}\,{{exams}}}\\ 	 \times \frac{{{number}}\,{{of}}\,{{exams}}\,{{with}}\,({{csPCa}}\cap {{PI}}\mbox{-}{{RADS}}\ge 3)}{{{number}}\,{{of}}\,{{exams}}\,{{with}}\,({{any}}\,{{pathological}}\,{{diagnosis}}\cap {{PI}}\mbox{-}{{RADS}}\ge 3)}\\ 	 \times \frac{{{number}}\,{{of}}\,{{exams}}\,{{with}}\,({{any}}\,{{pathological}}\,{{diagnosis}}\cap {{{PI}}}\mbox{-}{{RADS}}\ge 3)}{{{number}}\,{{of}}\,{{exams}}\,{{with}}\,{{PI}}\mbox{-}{{RADS}}\ge 3}\\ = 	 \,{{AIR}} \times {{PPV}}\,{{at}}{{PI}}\mbox{-}{{RADS}}\ge 3\times {{Pathological}}\,{{confirmation}}\,{{rate}}\,{{at}}\,{{PI}}\mbox{-}{{RADS}}\ge 3$$

$${{CDR}}\,{{ratio}}= 	 \frac{{{CDR}}\,({{target}})}{{{CDR}}\,({{control}})} =\frac{{{AIR}}\,({{target}})}{{{AIR}}\,({{control}})}\\ 	 \times \frac{{{PPV}}\, {{at}}\, {{PI}}-{{RADS}}\ge 3\,({{target}})}{{{PPV}}\, {{at}}\, {{PI}}-{{RADS}}\ge 3\,({{control}})}\\ 	 \times \frac{{{Pathological}}\,{{confirmation}}\,{{rate}}\,{{at}}\,{{PI}}\mbox{-}{{RADS}}\ge 3\,({{target}})}{{{Pathological}}\,{{confirmation}}\,{rate}\,{{at}}\,{{PI}}\mbox{-}{RADS}\ge 3\,({{control}})}\\ = 	 {{AIR}}\,{{ratio}}\times {{PPV}}\,{{ratio}}\times {{Pathological}}\,{{confirmation}}\,{rate}\,{{ratio}}$$

#### Secondary analysis

Secondary analysis evaluated the above metrics and their ratios using the following subgroups in order to investigate the impacts of gas-induced susceptibility artifacts by tumor location (peripheral zone and transition zone). The subgroups were created from the target groups with mild, moderate, and severe artifact grades and the corresponding control groups, respectively. Subgroup with the peripheral (transition) zone tumor was created by excluding examinations that described the transitional (peripheral) zone as the dominant lesion location and had pathologically proven csPCa.

In addition, factors associated with IQ were evaluated. For simplicity, IQ was binarized (severe–moderate vs mild–optimal). Examinations included in the initial DL dataset were used for the radiologists’ assessments; those not included were for the model.

All metrics were calculated at the examination level using Python 3.11. The chi-squared test was used with an alpha level of 0.05.

## Results

### IQ classification model

#### DL dataset

**Fig. 4:**
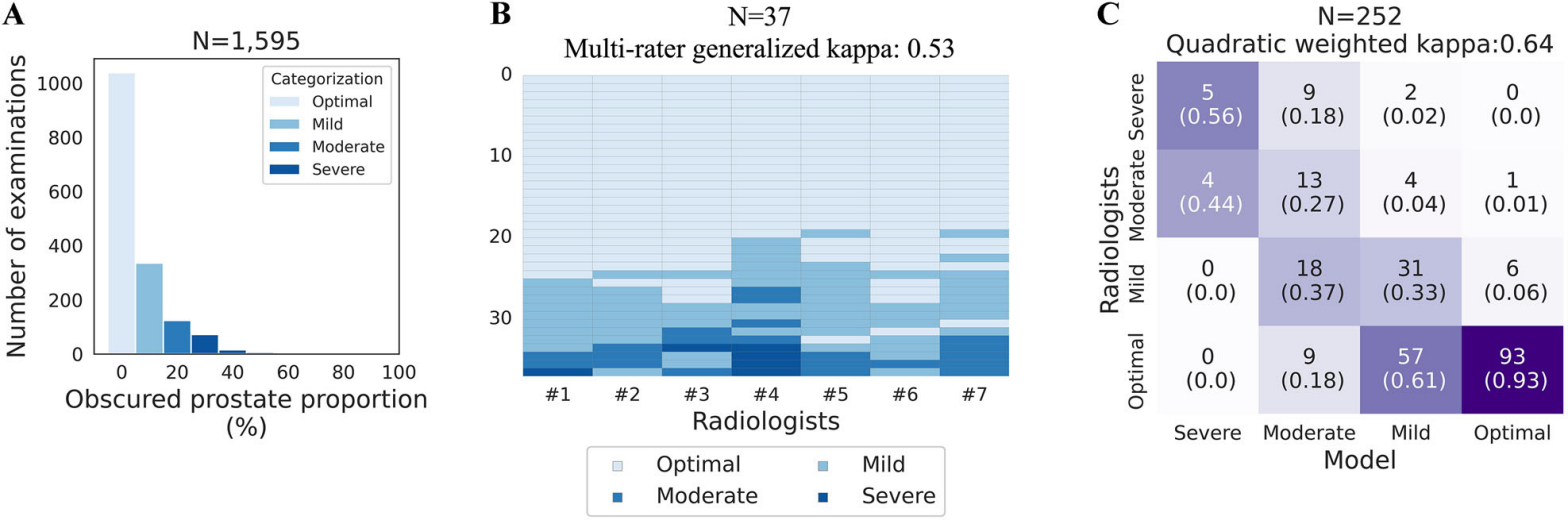
Image quality (IQ) assessments in the DL dataset. **A** Distribution of the obscured prostate proportion. The *x*-axis represents the median of the obscured prostate proportions categorized by radiologists; the y-axis represents the number of examinations. **B** Inter-reader variability among radiologists. The *x*-axis represents the radiologists; the *y*-axis represents the examinations reviewed by all radiologists in the test set. The cells indicate the degree of susceptibility artifacts assigned by radiologists. The darker the color, the worse the IQ. Inter-reader variability was assessed using multi-rater generalized kappa, shown in the title. **C** Confusion matrix of the model’s predictions against radiologists’ assessments. Each row represents the degree of susceptibility artifacts categorized by radiologists, whereas each column represents those by models. Each cell indicates the number of examinations with proportions over the model’s prediction in parentheses. DL, deep learning

Table 1 shows the details of the development set and test set. Of the 1595 total examinations, 1039 (65.1%) were categorized as optimal, 335 (21.0%) as mild, 135 (8.5%) as moderate, and 96 (6.0%) as severe. Figure 4A shows the distribution of obscured prostate proportions. In the 96 examinations categorized as severe (i.e., obscured prostate proportions ≥ 30%), 75 (78.1%) had obscured prostate proportions of 30–40%. Both the development and test sets had no significant difference in the distribution of the PI-RADS score, facility, and examination year across IQ classes (Table S1).

**Table 1:** DL dataset

		Development	Test	*p*
*N*		1343 (84.2%)	252 (15.8%)	
Age (years)*		66.0 ± 8.4	65.9 ± 8.8	0.87
Facility	I	717 (53.4%)	139 (55.2%)	0.79
	II	334 (24.9%)	63 (25.0%)	
	III	292 (21.7%)	50 (19.8%)	
Number of reviewers	1	1343 (100.0%)		**< 0.001**
	2		104 (41.3%)	
	3		111 (44.0%)	
	7		37 (14.7%)	
Year	2021	370 (27.6%)	60 (23.8%)	0.08
	2020	299 (22.3%)	49 (19.4%)	
	2019	272 (20.3%)	48 (19.0%)	
	2018	242 (18.0%)	50 (19.8%)	
	2017	160 (11.9%)	45 (17.9%)	
Magnetic field strength	3 T	1327 (98.8%)	249 (98.8%)	1.00
	1.5 T	16 (1.2%)	3 (1.2%)	
Manufacture	GE	703 (52.3%)	137 (54.4%)	0.60
	SIEMENS	640 (47.7%)	115 (45.6%)	
Scanner	DISCOVERY MR750w	637 (47.4%)	124 (49.2%)	0.87
	Skyra	511 (38.0%)	95 (37.7%)	
	MAGNETOM Vida	122 (9.1%)	19 (7.5%)	
	Others	73 (5.4%)	14 (5.6%)	
Repetition time (ms)^**^		4900 [4500–5980]	4812 [4271–5943]	0.18
Echo time (ms)^**^		69.2 [64.9–81.0]	71.1 [65.0–83.0]	0.14
FOV (RL, mm)^**^		110 [110–199]	112 [110–199]	0.38
FOV (AP, mm)^**^		220 [168–220]	220 [168–220]	0.58
Acquisition matrix size^**^	Phase-encoding direction	64 [64–72]	64 [64–72]	0.21
	Frequency-encoding direction	128 [126–134]	128 [126–134]	0.57
Pixel size (mm)^**^		1.7 [1.6–1.7]	1.7 [1.6–1.7]	0.89
Slice thickness (mm)^**^		3.0 [3.0–4.0]	3.0 [3.0–4.0]	0.16
PI-RADS	1–2	700 (52.1%)	136 (54.0%)	0.96
	3	166 (12.4%)	30 (11.9%)	
	4	279 (20.8%)	50 (19.8%)	
	5	198 (14.7%)	36 (14.3%)	
Minimum *b*-value (s/mm²)	100	510 (38.0%)	108 (42.9%)	0.22
	50	595 (44.3%)	97 (38.5%)	
	0	238 (17.7%)	47 (18.7%)	
IQ	Optimal	880 (65.5%)	159 (63.1%)	0.89
	Mild	280 (20.8%)	55 (21.8%)	
	Moderate	103 (7.7%)	22 (8.7%)	
	Severe	80 (6.0%)	16 (6.3%)	

Unless otherwise specified, data are the number of examinations with percentages in parentheses. The chi-squared test was used for comparison

*p*-values less than 0.05 are considered statistically significant and are shown in bold

*AP* anteroposterior, *csPCa* clinically significant prostate cancer, *DL* deep learning, *FOV* field of view, *PI-RADS* prostate imaging-reporting and data system, *RL* right–left

* Data are means with standard deviations. The Student *t*-test was used for comparison

** Data are medians, with interquartile ranges in square brackets. The Wilcoxon rank sum test was used for comparison

#### Evaluation on the test set

Inter-reader agreement among the seven radiologists in the 37 cases was moderate, with a multi-rater generalized kappa of 0.53 (Fig. 4B). Figure 4C shows the confusion matrix of the models’ predictions against radiologists’ assessments. The agreement was substantial, with a quadratic weighted kappa of 0.64. In 9 cases that the model categorized as severe, radiologists categorized 5 (56%) examinations as severe and 4 (44%) as moderate. In 100 cases that the model categorized as optimal, radiologists categorized 93 (93%) examinations as optimal and 6 (6%) as mild.

### CDR comparison

#### Population

For the included 12,947 examinations (13,707 DWI series) performed with clinical suspicion of csPCa, representative overall statistics were as follows: patient age, 65.5 ± 8.4 (mean ± standard deviation); proven csPCa, 3234 (25.0%) examinations; AIR, 0.45 (5790/12,947); CDR, 0.24 (3065/12,947). Prostate-specific antigen values and prostate volume were missing in 2291 (17.7%) and 788 (6.1%) examinations, respectively.

Of 12,594 DWI series to which the IQ classification model was applied, 100 (0.8%) were categorized as severe, 1238 (9.8%) as moderate, 4615 (36.6%) as mild, and 6641 (52.7%) as optimal. Radiologists reviewed all 100 series categorized as severe and 200 as moderate. Figure S1 shows the confusion matrix of the model’s predictions against the radiologists’ assessments.

#### Matching

After selecting the best examination-level IQ, 632 examinations from 617 men (mean age, 66.0 ± 9.5 [standard deviation]) were included as the target group: 141 (22.3%) severe, 161 (25.5%) moderate, and 330 (52.2%) mild examinations. For each, 705, 966, and 1320 matched control examinations with optimal IQ were selected from 7046 control pool examinations by 1:5, 1:6, and 1:4 matching, respectively.

Table 2 shows the patients’ characteristics of potential confounding factors before and after adjustment. After adjustment, the standardized mean differences of all confounding factors were less than 0.10, which indicates a good balance of covariate distribution between groups [30]. Table S2 shows the post-MRI patients’ characteristics before and after the same adjustment. After adjusting for potential confounding factors, no significant difference was seen in the csPCa proportion between the target and matched control groups.

**Table 2:** Potential confounding factors of clinically significant prostate cancer before and after adjustment with case-control matching

Unadjusted analysis				Adjusted analysis			
Artifact severity	Potential confounding factors	Target group	Control pool	SMD	Target group	Matched control	SMD
Severe	*N*	141	7046		141	705	
	Age (mean ± SD)	67.0 ± 10.1	65.3 ± 8.2	0.18	67.0 ± 10.1	66.6 ± 9.0	**0.05**
	PSA (median [IQR])	5.6 [3.6–9.1]	6.3 [4.6–9.0]	**0.01**	5.6 [3.6–9.1]	5.7 [3.9–8.6]	**0.09**
	Facility						**< 0.001**
	I	64 (45.4%)	3740 (53.1%)	0.80^**^	64 (45.4%)	320 (45.4%)	
	II	71 (50.4%)	1514 (21.5%)		71 (50.4%)	355 (50.4%)	
	III	6 (4.3%)	1792 (25.4%)		6 (4.3%)	30 (4.3%)	
	Biopsy status						**< 0.001**
	Naive	47 (33.3%)	2169 (30.8%)	0.21	47 (33.3%)	235 (33.3%)	
	Prev benign	30 (21.3%)	2126 (30.2%)		30 (21.3%)	150 (21.3%)	
	Unknown	64 (45.4%)	2751 (39.0%)		64 (45.4%)	320 (45.4%)	
Moderate	*N*	161	7046		161	966	
	Age (mean ± SD)	65.7 ± 10.3	65.3 ± 8.2	**0.04**	65.7 ± 10.3	65.8 ± 9.2	**−0.01**
	PSA (median [IQR])	5.7 [4.3–8.2]	6.3 [4.6–9.0]	−0.09^*^	5.7 [4.3–8.2]	5.8 [4.1–8.6]	**−0.08**
	Facility						**< 0.001**
	I	80 (49.7%)	3740 (53.1%)	0.36^**^	80 (49.7%)	480 (49.7%)	
	II	57 (35.4%)	1514 (21.5%)		57 (35.4%)	342 (35.4%)	
	III	24 (14.9%)	1792 (25.4%)		24 (14.9%)	144 (14.9%)	
	Biopsy status						**< 0.001**
	Naive	58 (36.0%)	2169 (30.8%)	0.17	58 (36.0%)	348 (36.0%)	
	Prev benign	37 (23.0%)	2126 (30.2%)		37 (23.0%)	222 (23.0%)	
	Unknown	66 (41.0%)	2751 (39.0%)		66 (41.0%)	396 (41.0%)	
Mild	*N*	330	7046		330	1,320	
	Age (mean ± SD)	65.8 ± 8.7	65.3 ± 8.2	**0.05**	65.8 ± 8.7	65.8 ± 8.3	**0.00**
	PSA (median [IQR])	6.0 [4.5–8.4]	6.3 [4.6–9.0]	**−0.08**	6.0 [4.5–8.4]	5.9 [4.5–8.3]	**−0.05**
	Facility						**< 0.001**
	I	168 (50.9%)	3740 (53.1%)	0.33^**^	168 (50.9%)	672 (50.9%)	
	II	111 (33.6%)	1514 (21.5%)		111 (33.6%)	444 (33.6%)	
	III	51 (15.5%)	1792 (25.4%)		51 (15.5%)	204 (15.5%)	
	Biopsy status						**< 0.001**
	Naive	80 (24.2%)	2169 (30.8%)	0.19^**^	80 (24.2%)	320 (24.2%)	
	Prev benign	91 (27.6%)	2126 (30.2%)		91 (27.6%)	364 (27.6%)	
	Unknown	159 (48.2%)	2751 (39.0%)		159 (48.2%)	636 (48.2%)	

Unless otherwise specified, data are the number of examinations, with or without percentages in parentheses. Unadjusted and adjusted analyses represent the statistics before and after the coarsened exact matching. The standardized mean difference (SMD) less than 0.10 indicates a good balance of covariate distribution between the groups and is shown in bold

The mean of the patient age was compared using the Student's *t*-test. The median of the PSA value was compared using the Wilcoxon rank sum test. Other variables were compared using the chi-squared test

*IQR* interquartile range, *Prev benign* previous history of benign prostate biopsy, *PSA* prostate-specific antigen, *SD* standard deviation

* *p*-values less than 0.05

** *p*-values less than 0.01

#### Primary analysis

**Fig. 5:**
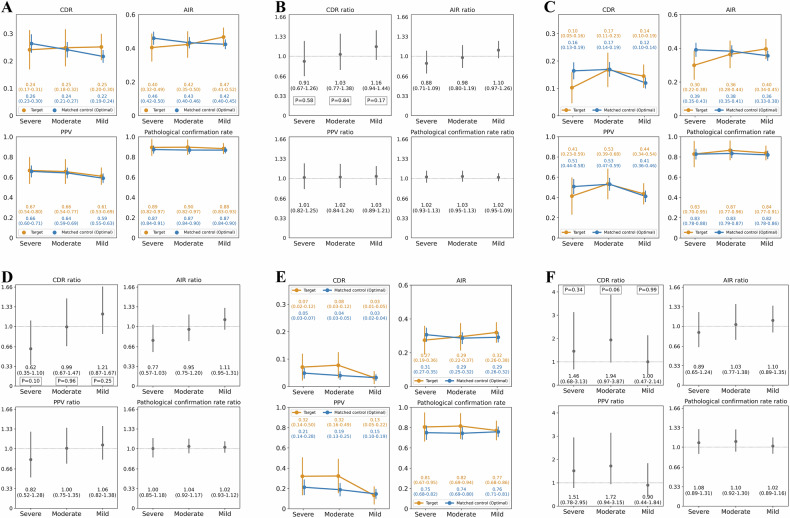
Diagnostic performance by degree of gas-induced susceptibility artifacts in diffusion-weighted images. **A**, **B** Entire target group and matched control group. **C**, **D** Subgroups from the target group and the matched control group, focusing on the peripheral zone tumor. **E**, **F** Subgroups from the target group and the matched control group, focusing on the transition zone tumor. **A**, **C**, **E** Comparisons between the target groups and matched control groups. The orange and blue plots represent the diagnostic performance of the target group and the corresponding matched control groups, respectively, with 95% confidence intervals (CIs) in the vertical lines. The color text in each column represents the corresponding color plots’ values with 95% CIs in parentheses. **B**, **D**, **F** Diagnostic performance ratios. The y-axis represents the ratio of diagnostic performance, calculated by dividing the diagnostic performance in the target group by that in the corresponding matched control group. The vertical lines represent the 95% CIs of the ratio. The exact values and 95% CIs in parentheses are written below each plot. For the CDR ratio, *p*-values were calculated for each IQ class using the chi-squared test and are shown at the bottom. AIR, abnormal interpretation rate; CDR, cancer detection rate; PPV, positive predictive value

Figure 5A, B show point plots indicating the diagnostic performance by the degree of susceptibility artifacts. The CDR in the target groups and the corresponding matched control groups were, respectively, as follows: severe vs optimal, 0.24 vs 0.26, CDR ratio = 0.91 [95% confidence interval: 0.67–1.26], *p* = 0.58; moderate vs optimal, 0.25 vs 0.24, CDR ratio = 1.03 [0.77–1.38], *p* = 0.84; mild vs optimal, 0.25 vs 0.22, CDR ratio = 1.16 [0.94–1.44], *p* = 0.17. In the severe cases, the AIR ratio (0.88) was the lowest, followed by the biopsy rate ratio (1.01) and PPV ratio (1.02).

#### Secondary analysis

**Fig. 6:**
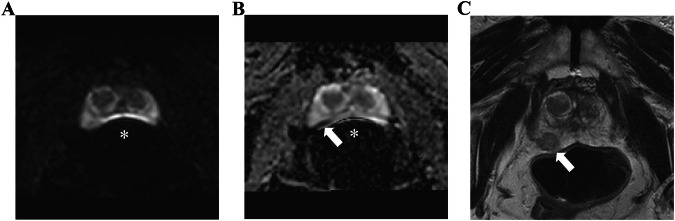
Severe gas-induced susceptibility artifacts that obscured csPCa in diffusion-weighted image (DWI). **A** DWI (*b*-value 0 s/mm²). **B** Apparent diffusion coefficient (ADC) map. **C** T2-weighted image (T2WI). An 83-year-old man with elevated prostate-specific antigen values of 12.2 ng/mL. Severe gas-induced susceptibility artifacts (*) are seen on DWI, with the obscure prostate proportion over 30%. No detectable lesion was found with DWI. Although an unreliable finding, the ADC map shows a suspiciously low signal intensity lesion at the right posterior peripheral zone. A focal hypointense lesion is present at the right posterior peripheral zone with focal bulging on the T2WI (arrow). Note the horizontally long field of view in the DWI and ADC map, which was scanned using an anteroposterior phase-encoding direction. PI-RADS 5 was assigned, and clinically significant prostate cancer was pathologically proven (Grade group 4)

Figure 5C–F are point plots, similar to Fig. 5A, B, but focused on the peripheral and transitional zone tumors, respectively. Regarding the peripheral zone tumor, the CDR ratio decreased as the degree of gas-induced susceptibility artifact increased, although no statistical significance was seen even in severe artifact cases (severe vs optimal, 0.10 vs 0.16, CDR ratio = 0.62, *p* = 0.10; moderate vs optimal, 0.17 vs 0.17, CDR ratio = 0.99, *p* = 0.96; mild vs optimal, 0.14 vs 0.12, CDR ratio = 1.21, *p* = 0.25). Figure 6 shows an example with csPCa in the peripheral zone, which was obscured by severe gas-induced susceptibility artifacts in DWI but was assigned a PI-RADS 5 based on the T2WI finding. In contrast, no reduction was seen in the CDR ratio in the transition tumor (severe vs optimal, 0.07 vs 0.05, CDR ratio = 1.46, *p* = 0.34; moderate vs optimal, 0.08 vs 0.04, CDR ratio = 1.94, *p* = 0.06; mild vs optimal, 0.03 vs 0.03, CDR ratio = 1.00, *p* = 0.99).

Table 3 shows the patient-related factors associated with IQ. The proportion of Facility II was significantly higher in the poor-quality series than that in the mild–optimal series (radiologists: 35.6% vs 24.3%, *p* < 0.001; model: 41.1% vs 25.9%, *p* < 0.001). The prostate volume was significantly smaller in the poor-quality series than that in the mild–optimal series (radiologists: 46.0 mL vs 53.0 mL, *p* = 0.005; model: 45.0 mL vs 54.0 mL, *p* < 0.001). The presence of csPCa was not significantly associated with IQ.

**Table 3:** Association between patient-related factors and binarized degree of susceptibility artifacts from rectal gas

		Radiologists (initial DL dataset)			Model		
		Severe–moderate	Mild–optimal	*p*	Severe–moderate	Mild–optimal	*p*
*N*		132	642		1338	11,256	
Age^*^		66.5 ± 9.5	65.4 ± 8.2	0.21	66.1 ± 8.8	65.5 ± 8.2	**0.02**
Facility	I	75 (56.8%)	347 (54.0%)	**< 0.001**	694 (51.9%)	5757 (51.1%)	**< 0.001**
	II	47 (35.6%)	156 (24.3%)		550 (41.1%)	2917 (25.9%)	
	III	10 (7.6%)	139 (21.7%)		94 (7.0%)	2582 (22.9%)	
PI-RADS	1–2	67 (50.8%)	366 (57.0%)	0.37	729 (54.5%)	6273 (55.7%)	0.34
	3	12 (9.1%)	68 (10.6%)		140 (10.5%)	1235 (11.0%)	
	4	32 (24.2%)	119 (18.5%)		278 (20.8%)	2107 (18.7%)	
	5	21 (15.9%)	89 (13.9%)		191 (14.3%)	1641 (14.6%)	
Prostate volume (cc)^**^		46.0 [33.2–61.0]	53.0 [38.0–77.0]	**0.005**	45.0 [34.0–63.5]	54.0 [38.0–80.0]	**< 0.001**
csPCa		50 (37.9%)	170 (26.5%)	**0.01**	357 (26.7%)	2770 (24.6%)	0.10

Unless otherwise specified, data are the number of examinations, with or without percentages in parentheses. The chi-squared test was used for comparison

*p*-values less than 0.05 are considered statistically significant and are shown in bold

*csPCa* clinically significant prostate cancer, *DL* deep learning, *PI-RADS* prostate imaging-reporting and data system

* Data are means with standard deviations. The Student's *t*-test was used for comparison

** Data are medians, with interquartile ranges in square brackets. The Wilcoxon rank sum test was used for comparison

Table 4 compares the inter-facility difference in scanning parameters using examinations in the DL dataset. Facility II used an anteroposterior phase-encoding direction more frequently than other facilities (99.7% vs 0.0–1.7%, respectively).

**Table 4:** Inter-facility difference in scanning parameters in diffusion-weighted imaging

		Facility I	Facility II	Facility III	*p*
*N*		708	438	231	
Manufacture	GE	696 (98.3%)	0 (0.0%)	0 (0.0%)	**< 0.001**
	SIEMENS	12 (1.7%)	438 (100.0%)	231 (100.0%)	
Magnetic field strength	3 T	703 (99.3%)	435 (99.3%)	229 (99.1%)	0.96
	1.5 T	5 (0.7%)	3 (0.7%)	2 (0.9%)	
Use of endorectal coil		30 (4.2%)	0 (0.0%)	0 (0.0%)	**< 0.001**
Phase encoding direction	RL	695 (98.2%)	1 (0.2%)	231 (100.0%)	**< 0.001**
FOV (mm)^*^	AP	220 [220–261]	169 [95–169]	220 [219–220]	**< 0.001**
	RL	110 [110–130]	200 [199–200]	98 [98–116]	**< 0.001**
Acquisition matrix size^*^	Phase-encoding direction	64 [64–72]	60 [60–98]	61 [60–68]	**< 0.001**
	Frequency-encoding direction	128 [128–144]	126 [116–126]	134 [128–134]	**< 0.001**
Pixel size (mm²)^*^		3.0 [3.0–3.3]	2.5 [2.5–3.0]	2.7 [2.7–3.2]	**< 0.001**
Slice thickness (mm)^*^		3.0 [3.0–4.0]	3.0 [3.0–3.0]	3.0 [3.0–4.0]	**< 0.001**
Low and intermediate *b*-values (s/mm²)	100–800	255 (36.0%)	0 (0.0%)	30 (13.0%)	**< 0.001**
	50–800	231 (32.6%)	102 (23.3%)	171 (74.0%)	
	100–1000	129 (18.2%)	0 (0.0%)	0 (0.0%)	
	0–800	0 (0.0%)	336 (76.7%)	12 (5.2%)	
	Others	93 (13.1%)	0 (0.0%)	18 (7.8%)	

Unless otherwise specified, data are the number of examinations, with or without percentages in parentheses. The chi-squared test was used for comparison

*p*-values less than 0.05 are considered statistically significant and are shown in bold

*AP* anteroposterior, *FOV* field of view, *RL* right–left

* Data are medians, with interquartile ranges in square brackets. The Kruskal–Wallis test was used for comparison

## Discussion

This study compared the CDR of prostate MRI by the degree of gas-induced susceptibility artifacts in DWI. The DL algorithms assisted radiologists in finding relatively uncommon cases with severe artifacts. No significant CDR reduction was seen, even in the examinations with severe artifacts, but the observed CDR modestly decreased as the degree of artifacts increased. Location-based analysis revealed that CDR reduction was seen especially in the peripheral zone tumor.

Previous studies on gas-induced artifacts have primarily focused on the relationship between rectal preparation and IQ, rather than diagnostic outcomes [31–34]. Wang et al evaluated the diagnostic impact and reported a tendency for reduced diagnostic performance as artifact severity increased, measured by the area under the curve [35]. However, this was not statistically significant. Our findings are consistent with this trend. In addition, we further demonstrated that the peripheral zone is particularly vulnerable to gas-induced artifacts, which is clinically plausible because it lies adjacent to the rectum and is therefore more frequently obscured.

Given that hip prostheses with obscured proportions greater than 33% can significantly reduce CDR [13], we initially anticipated a more pronounced detrimental effect of rectal gas. This expectation was backed by the observation that gas-related artifacts were more common than those caused by metallic implants. Additionally, approximately 80% of csPCa originates in the peripheral zone [14, 15]. Nevertheless, no statistically significant reduction in CDR was observed in our cohort. One possible explanation is that T2WI combined with dynamic contrast-enhanced imaging complements a csPCa diagnosis. In cases without appropriate DWI, PI-RADS recommends alternative use of T2WI as the dominant sequence for the peripheral zone [11]. A previous study showed that the degree of susceptibility artifact was weaker in T2WI compared to that in DWI [36]. Another possible explanation is that a relatively smaller obscured prostate proportion occurs due to gas as opposed to hip prostheses. Even though the thresholds of severe gas-induced artifacts (over 30%) in the current study and moderate-severe artifacts from hip prosthesis (over 33%) are similar, the average obscured volume in these groups was much greater with a hip prosthesis. Often, the hip prostheses obscured over 50% of the gland, whereas gas-induced artifacts rarely obscured to that degree.

The recently revised PI-QUAL v2 [37] evaluates the IQ of DWI using four items, including the presence of significant artifacts within the prostate. However, the definition of significant artifacts is subjective and unclear. As the susceptibility artifact is the main artifact in DWI, we proposed using the numerical obscured proportion to define a significant artifact. The width of susceptibility artifacts could be an alternative criterion [38], but this is difficult to apply to artifacts from hip prostheses. With this criterion, inter-reader agreement among radiologists was moderate and considered within the expected range. Previous studies on PI-QUAL reported fair-substantial agreements in DWI, with kappa statistics of 0.23–0.70 [9, 10, 31, 39, 40]. In the current study, the kappa statistic between the models and radiologists was not worse than that among radiologists. Our findings may provide additional insights for future refinements of PI-QUAL. First, the obscured proportion could serve as a more quantitative and objective criterion for defining significant artifacts, thereby reducing subjectivity in PI-QUAL scoring. Furthermore, because the diagnostic impact of artifacts varies according to their type and severity, a more refined framework that explicitly accounts for these differences may be considered.

The absence of serious prediction bias is a prerequisite when comparing diagnostic performance. The main performance metric of CDR is influenced by the csPCa proportion, PI-RADS assignment, and pathological confirmation rate. Of these, the most problematic bias will be the difference in the csPCa proportion among the different artifact groups. This is because IQ should be independent of the presence of csPCa, but it could affect the PI-RADS assignments and indications of prostate biopsy [9, 10]. To minimize the model’s prediction bias other than IQ, we adjusted the breakdown of the PI-RADS score and facility in the DL dataset. As a result, no significant association was found between the model’s prediction and the presence of csPCa. This indicated no serious prediction bias when comparing CDR.

Facilities were significantly associated with IQ. Among inter-facility differences in MRI acquisition parameters, the most relevant one seems to be the phase-encoding direction. The anteroposterior phase-encoding direction was used for most DWI series in Facility II, with a higher proportion of severe artifacts. In contrast, the right–left direction was used most often in the other facilities, where the proportions of severe artifacts were lower. Since DWI susceptibility artifacts are more prominent in the phase-encoding direction [12], using the anteroposterior direction is disadvantageous for rectal-induced artifacts.

Another major inter-facility difference was rectal preparation protocols. Facility III, with a relatively lower proportion of severe artifacts, instructed patients to go to the restroom to empty their rectum before scanning. An empty rectum is desirable because stool and rectal dilatation negatively impact susceptibility artifacts in prostate MRI [31, 41]. Currently, there is no consensus about optimal rectal preparation, and PI-RADS v2.1 advises “minimal” preparation [32]. However, various preparation protocols have been proposed after PI-RADS v2.1 [31–34]. Our revealed clinical impact of gas-induced artifacts will be one of the considerations in implementing those protocols. As a non-invasive and unburdened method, our findings suggest that instructing defecation before scanning and using right–left phase-encoding directions may be effective against gas-induced artifacts.

Our study had several limitations. First, the image qualities of DWI and other sequences were considered independent, and their combined impact was not evaluated. We assumed that gas-induced artifacts in T2WI or dynamic contrast-enhanced imaging have limited impacts on CDR because the degree of gas-induced artifacts in those sequences are less significant than in DWI. We also ignored the impacts of other IQ-related factors, such as motion, with an assumption that they are independent of gas-induced artifacts. Second, there was a potential bias in the radiologists’ assessments after the models’ categorization, which may overestimate the agreement between the model and radiologists. Finally, most images in the optimal quality group were not reviewed, although radiologists agreed with 93% of the examinations predicted as optimal in the test set. Future studies need to evaluate the impact of different rectal preparation protocols on CDR to determine the most appropriate protocol.

In summary, no statistical evidence was found that gas-induced DWI susceptibility artifacts degraded the CDR of prostate MRI. This may have resulted from the other sequences that complement the diagnosis of csPCa and the relatively smaller obscuration effects on the prostate.

## Supplementary information

ELECTRONIC SUPPLEMENTARY MATERIAL

## Abbreviations

AIR: Abnormal interpretation rate
CDR: Cancer detection rate
csPCa: Clinically significant prostate cancer
DL: Deep learning
DWI: Diffusion-weighted imaging
IQ: Image quality
PI-QUAL: Prostate image quality
PI-RADS: Prostate imaging-reporting and data system
PPV: Positive predictive value
T2WI: T2-weighted image
