# Supplementary material for: Prostate MRI cancer detection rate by deep learning-assisted image quality categorization: gas-induced susceptibility artifacts in diffusion-weighted imaging
Source: Insights Imaging. 2025 Oct 15;16:217. doi: 10.1186/s13244-025-02110-6 (PMC12528626; doi:10.1186/s13244-025-02110-6)
Supplement: Supplementary file 1 — ELECTRONIC SUPPLEMENTARY MATERIAL [file 13244_2025_2110_MOESM1_ESM.pdf]

Prostate MRI cancer detection rate by deep learning-assisted image quality categorization: Gas-induced susceptibility artifacts in diffusion-weighted imaging

Appendix S1

Model development

A deep learning model to categorize the degree of gas-induced susceptibility artifacts was developed using the development set. The 3D EfficientNet-B0 architecture [1] was used. MONAI 1.3.0 (https://monai.io/) and PyTorch 2.1.0 (https://pytorch.org/) were used with the following settings: loss function, ordinal cross-entropy; optimizer, Adam; the number of epochs, 35; learning rate, starts at 5e-3 and ends at 1e-4 using a cosine annealing schedule; batch size, 4. The PyTorch WeightedRandomSampler class was used to mitigate the class imbalance of the artifact severity (oversampling of poor-quality series). Five different models were developed through five-fold stratified cross-validation by the facility, examination year, and presence of clinically significant prostate cancer.

Reference

1. Tan M, Le Q (09--15 Jun 2019) EfficientNet: Rethinking Model Scaling for Convolutional Neural Networks. In: Chaudhuri K, Salakhutdinov R (eds) Proceedings of the 36th International Conference on Machine Learning. PMLR, pp 6105–6114

Table S1: Breakdown of the PI-RADS score, facility, and examination year by the degree of gas-induced susceptibility artifacts in diffusion-weighted imaging

			Severe	Moderate	Mild	Optimal	All	P-value
**Development set**	**PI-RADS**	**1–2**	41 (51.2%)	53 (51.5%)	146 (52.1%)	460 (52.3%)	700 (52.1%)	0.14
		**3**	6 (7.5%)	6 (5.8%)	35 (12.5%)	119 (13.5%)	166 (12.4%)	
		**4**	19 (23.8%)	21 (20.4%)	65 (23.2%)	174 (19.8%)	279 (20.8%)	
		**5**	14 (17.5%)	23 (22.3%)	34 (12.1%)	127 (14.4%)	198 (14.7%)	
	**Facility**	**I**	43 (53.8%)	52 (50.5%)	150 (53.6%)	472 (53.6%)	717 (53.4%)	0.052
		**II**	30 (37.5%)	25 (24.3%)	70 (25.0%)	209 (23.8%)	334 (24.9%)	
		**III**	7 (8.8%)	26 (25.2%)	60 (21.4%)	199 (22.6%)	292 (21.7%)	
	**Year**	**2017**	9 (11.2%)	12 (11.7%)	47 (16.8%)	92 (10.5%)	160 (11.9%)	0.06
		**2018**	14 (17.5%)	19 (18.4%)	56 (20.0%)	153 (17.4%)	242 (18.0%)	
		**2019**	14 (17.5%)	21 (20.4%)	41 (14.6%)	196 (22.3%)	272 (20.3%)	
		**2020**	20 (25.0%)	18 (17.5%)	52 (18.6%)	209 (23.8%)	299 (22.3%)	
		**2021**	23 (28.8%)	33 (32.0%)	84 (30.0%)	230 (26.1%)	370 (27.6%)	
**Test set**	**PI-RADS**	**1–2**	7 (43.8%)	13 (59.1%)	33 (60.0%)	83 (52.2%)	136 (54.0%)	0.42
		**3**	4 (25.0%)	0 (0.0%)	7 (12.7%)	19 (11.9%)	30 (11.9%)	
		**4**	3 (18.8%)	7 (31.8%)	9 (16.4%)	31 (19.5%)	50 (19.8%)	
		**5**	2 (12.5%)	2 (9.1%)	6 (10.9%)	26 (16.4%)	36 (14.3%)	
	**Facility**	**I**	10 (62.5%)	15 (68.2%)	30 (54.5%)	84 (52.8%)	139 (55.2%)	0.58

	**II**	4 (25.0%)	6 (27.3%)	14 (25.5%)	39 (24.5%)	63 (25.0%)	
	**III**	2 (12.5%)	1 (4.5%)	11 (20.0%)	36 (22.6%)	50 (19.8%)	
**Year**	**2017**	5 (31.2%)	4 (18.2%)	16 (29.1%)	20 (12.6%)	45 (17.9%)	0.11
	**2018**	4 (25.0%)	6 (27.3%)	13 (23.6%)	27 (17.0%)	50 (19.8%)	
	**2019**	3 (18.8%)	2 (9.1%)	11 (20.0%)	32 (20.1%)	48 (19.0%)	
	**2020**	2 (12.5%)	5 (22.7%)	8 (14.5%)	34 (21.4%)	49 (19.4%)	
	**2021**	2 (12.5%)	5 (22.7%)	7 (12.7%)	46 (28.9%)	60 (23.8%)	

Data are the number of examinations, with or without percentages in parentheses. The chi-squared test was used for comparison. PI-RADS = Prostate Imaging-Reporting and Data System

Table S2: Post-MRI patients' findings before and after adjustment for potential confounding factors

		Unadjusted analysis			Adjusted analysis		
**Artifact severity**	**Post-MRI findings**	**Target group**	**Control pool**	**SMD**	**Target group**	**Matched control**	**SMD**
**Severe**	**N**	141	7,046		141	705	
	**PI-RADS**						0.12
	**1–2**	84 (59.6%)	3,851 (54.7%)	0.17	84 (59.6%)	381 (54.0%)	
	**3**	10 (7.1%)	830 (11.8%)		10 (7.1%)	62 (8.8%)	
	**4**	25 (17.7%)	1,326 (18.8%)		25 (17.7%)	139 (19.7%)	
	**5**	22 (15.6%)	1,039 (14.7%)		22 (15.6%)	123 (17.4%)	
	**Prostate volume (cc, median [IQR])**	38.0 [30.8–51.2]	54.0 [38.0–79.0]	-0.63 **	38.0 [30.8–51.2]	49.0 [35.0–75.0]	-0.53 **
	**csPCa**	36 (25.5%)	1,763 (25.0%)	0.01	36 (25.5%)	196 (27.8%)	0.05
**Moderate**	**N**	161	7,046		161	966	
	**PI-RADS**						0.24
	**1–2**	93 (57.8%)	3,851 (54.7%)	0.28 *	93 (57.8%)	548 (56.7%)	
	**3**	7 (4.3%)	830 (11.8%)		7 (4.3%)	99 (10.2%)	
	**4**	33 (20.5%)	1,326 (18.8%)		33 (20.5%)	179 (18.5%)	
	**5**	28 (17.4%)	1,039 (14.7%)		28 (17.4%)	140 (14.5%)	
	**Prostate volume (cc, median [IQR])**	43.0 [33.8–55.3]	54.0 [38.0–79.0]	-0.54 **	43.0 [33.8–55.3]	53.0 [36.0–78.0]	-0.50 **
	**csPCa**	42 (26.1%)	1,763 (25.0%)	0.02	42 (26.1%)	245 (25.4%)	0.02
**Mild**	**N**	330	7,046		330	1,320	
	**PI-RADS**						0.10

**1–2**	176 (53.3%)	3,851 (54.7%)	0.06	176 (53.3%)	761 (57.7%)	
**3**	40 (12.1%)	830 (11.8%)		40 (12.1%)	142 (10.8%)	
**4**	69 (20.9%)	1,326 (18.8%)		69 (20.9%)	238 (18.0%)	
**5**	45 (13.6%)	1,039 (14.7%)		45 (13.6%)	179 (13.6%)	
**Prostate volume (cc, median [IQR])**	47.0 [34.0–66.0]	54.0 [38.0–79.0]	-0.28 **	47.0 [34.0–66.0]	53.0 [36.0–78.1]	-0.27 **
**csPCa**	91 (27.6%)	1,763 (25.0%)	0.06	91 (27.6%)	300 (22.7%)	0.11

Unless otherwise specified, data are the number of examinations, with or without percentages in parentheses. The chi-squared test was used for comparison.
The median of the PSA value was compared using the Wilcoxon rank sum test.
Unadjusted and adjusted analyses represent the statistics before and after the coarsened exact matching.
The standardized mean difference (SMD) less than 0.10 indicates a good balance of covariate distribution between the groups. *P-values less than 0.05. **P-values less than 0.01.

csPCa = clinically significant prostate cancer, IQR = Interquartile range, PI-RADS = Prostate Imaging-Reporting and Data System

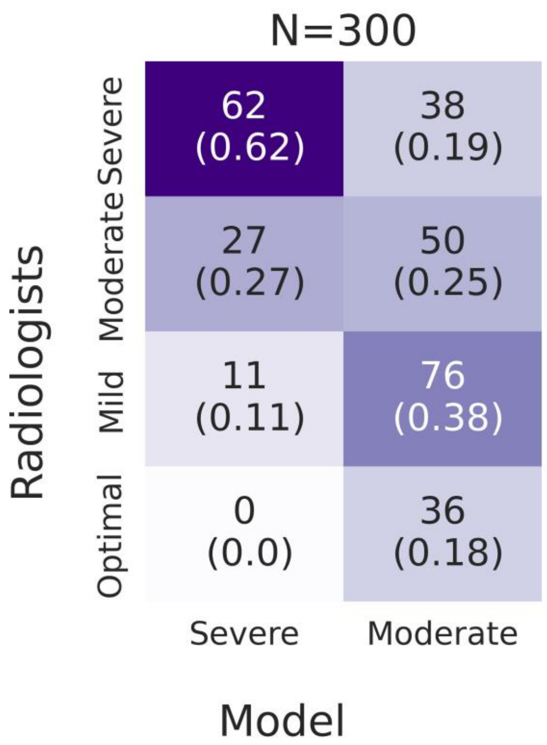

Figure S1: Confusion matrices of the radiologists' categorization against the models after their predictions

Each row represents the degree of susceptibility artifacts categorized by radiologists, whereas each column represents that by models. Each cell indicates the number of examinations reviewed by radiologists after the models' prediction, with proportions over the prediction in parentheses.
